# The smart body concept as a demonstration of the overarching utility and benefits of 3D avatars in retail, health and wellbeing: an accuracy study of body measures from 3D reconstruction

**DOI:** 10.1007/s11042-022-13661-x

**Published:** 2022-09-13

**Authors:** Veronica Cimolin, Ioannis Th. Paraskevopoulos, Maurizio Sala, Marco Tarabini, Manuela Galli

**Affiliations:** 1 Department of Electronics, Information and Bioengineering, Politecnico di Milano, Piazza Leonardo da Vinci 32, 20133 Milan, Italy; 2 Orthopedic Rehabilitation Unit and Research Laboratory in Biomechanics, Rehabilitation and Ergonomics, Istituto Auxologico Italiano, IRCCS, San Giuseppe Hospital, 28824 Piancavallo, Italy; 3 IGOODI SrL, Via Gaetano Negri, 4, 20123 Milan, Italy; 4 Department of Mechanical Engineering, Politecnico di Milano, via La Masa 1, 20156 Milan, Italy

**Keywords:** Avatar, Body scanner, Anthropometry, 3D models, Accuracy, Fashion, Fitness, Wellness, Healthcare

## Abstract

Recent developments in 3D graphic technologies enable the affordable and precise reconstruction of body scanned models that can be applied in a variety of verticals, such as fashion, fitness and wellness, and healthcare. The accuracy of body measurements is a crucial element for the successful application of avatars in the following use cases: Avatars that go beyond visual representation and offer intrinsic and precise anthropometric data defined as a smart body are discussed in this paper. In particular, this paper presents the Gate technology, an innovative, autonomous, sustainable body scanner, coupled with an automatic production pipeline and the concept of avatars as smart bodies. We present an accuracy study of scanning technology for scanning inanimate objects, as well as body parts versus the ground, by using an established accuracy scanning system. The results appear to be promising and confirm the hypothesis of applying the technology to the use cases discussed as well as broadening the research to other studies and future applications.

## Introduction

The recent pandemic emergency increased the necessity of digital transformation in not only areas, such as education and health, but also in everyday tasks, such as online shopping [20, 21, 31]. In this context, the growth of the apparel market [43] is limited by the restrictions imposed on physical retail activities [42, 45]. Retail digitalisation has grown immensely with business models shifting toward e-commerce and online shopping solutions [41]. However, one of the major technical issues to overcome to reach such a disruption is fitting and sizes. Correct and accurate sizing is a considerable problem for online clothes retailers that face levels of returned packages from 30% to even 50% of cases in certain vertical sectors and countries [11].

Such a high number of returns impedes the proliferation of online commerce solutions in the fashion and apparel industries, as it implies massive costs for the distribution and production of goods, which results in higher prices. Accurate anthropometric measurements can provide a perfect solution to the problem, and 3D technologies can encompass the visualization of the digital twin of the human body in the form of avatars in 3D graphics, while also becoming a container of personal data.

Such data include body measurements and other anthropometric data related to health, wellbeing, and personal preference. The concept of an avatar that is not restricted to 3D graphical visualization of the person but as an embodiment of data in the form of a digital online ID is described by the authors as the concept of the smart body. In this way, avatars are not limited to visualization purposes that in realistic contexts are bound to subjective acceptance due to the uncanny valley effect [36]; their applicability and usefulness can be extended as a utility asset containing personal data and information that is useful for performing tasks such as, for example, secure online shopping with accurate sizing and fitting, highly personalized offers and services. Avatars with utility (such as but not limited to digital identification) will play a major role and have considerable implications across platforms and, more specifically, in the fashion sector and selling virtual merchandise as an added value to the real product in the form of a nonfungible token (NFT) [17].

Other areas where smart bodies can be useful and provide solutions include health and well-being [13, 14], with immediate applications in the orthopedic sector since they allow 3D printing of braces that are very faithful to those of the patient’s body.

The creation of new habits, such as sports training at home, became a trend that emerged from the pandemic and boosted the online provision of personal training and nutrition consultancy to unprecedented heights [12]. This is another area where the concept of the smart body can create new paradigms for disruption and innovation utilizing the measurements of the human body, data about the person and visualizing the exact digital copy of the user in 3D. Precise body measurements allow for remote monitoring of the advancement of an exercising and diet regime over time.

Furthermore, the recent advances of the metaverse concept [39] along with the Web3 revolution [28] render avatars a necessary representation and identification medium to populate metaverses. The technology proposed in this paper is an autonomous, scalable, automatic body scanner (the Gate) coupled with an automatic production pipeline that can produce realistic scans of people with such a level of accuracy enough to satisfy the use cases of fashion, wellness and also potentially of healthcare. The technology is easily deployable on the territory and managed completely remotely. This study aims to demonstrate the scanning accuracy of the Gate, establishing the baseline for enabling derivative use cases and applications.

### Aim of the research

This paper describes an automatic process to capture anthropometric measurements and to enrich them by transforming avatars into smart bodies. The aim of this study is twofold: first, to assess the accuracy and precision of technology for a 3D reconstruction of the digital body by comparing between the proposed system and the LS3D method on a series of regularly shaped objects of known volume; the second aim of this study is to quantify and compare the linear measures, diameters and volumes of the human lower limbs assessed with the system described in this work and in LS3D.

### Background (literature review)

Section 1: Studies on state-of-the-art accurate body scanning

Currently, there are different techniques for measuring the human body, ranging from simple indirect measurements to more sophisticated direct volumetric measurements, such as video, visualization and 3D body scanning. Conventional manual methods of collecting body measurements using anthropometers, calipers and measuring tapes are simple and inexpensive. However, they have some limitations, such as (a) the time required for measurement, (b) the necessity of frequent equipment calibration and trained observers, (c) changes in the patient’s body posture, (d) variations in tape pressure during measurement, and (e) the identification of reference points, which are difficult for people with more body fat [6, 16].

At present, to overcome these limitations, advanced anthropometric measurement systems utilizing.

3D body-scanning technologies are having a growing impact on product development, production and consumption processes [18]. 3D body scanners can capture a human body or only specific parts [8, 9, 18, 33] to generate detailed 3D models within a few seconds. 3D body scanners are innovative in capturing, measuring and tracking the evolution of the human body in various fields, such as medical, fitness or entertainment. Initially, developed for the clothing industry [29, 30, 37], 3D body scanners are greatly improving the ability to measure and visualize a person’s body size and shape. Recent advances in whole-body scanning unlock new potential, especially for healthcare applications due to their noninvasiveness, rapidity and accuracy.

In addition to a 3D body scanner, to measure the volume of specific body segments, the laser scanner 3D method (LS3D) can be used. It is applied in orthopedics, on the design of orthoses, and for other health and well-being applications. It has the advantage of being relatively inexpensive, fast, accurate, noninvasive and requiring no contact with the patient [18]. LS3D has been studied in terms of accuracy and reproducibility and compared with the gold standard, i.e., water displacement (WD), demonstrating good results for small objects and specific body segments, such as upper and lower limbs [7–9, 33, 35]. A body of literature has already dealt with the creation of databases of scanned people as statistical populations of 3D human bodies [19, 40], addressing the sizing issue in fashion and the clusters of the population in terms of body shapes. There are also ISO standards developed in an attempt to standardize the way the industry applies anthropometric measurements and 3D scanning methods [1, 2]. There is a clear gap in the literature where body scans are used as demographic populations for statistical analyses and not as personalized representation and identification for the user themselves. Examples of such use cases include sizing and made-to-measure in Fashion, personalized nutrition, training prescription and monitoring in health, postural analyses, mole inventory, etc. A fundamental element of these use cases is accurately capturing the measurements in a scalable manner.

Section 2: Studies on state-of-the-art body shape analysis in fashion

Body shape analysis is one of the earliest techniques adopted for the classification of biological specimens [25]. More recently, researchers have made numerous attempts to analyze and classify the human body in efforts to improve clothing fits and the sizing system [51]. Traditional analyses utilized manual 2D measurement data and photographic images that have been used to study obesity, gender evaluation of physical attractiveness, and body image. Gazzuolo et al. [15] used statistical regression models to compare traditional linear measurements with measurements taken from frontal and lateral view photographs for use when developing patterns for the upper torso of female body forms. The research suggested the photographic method of measurement extraction using video capture and automated measuring of silhouette angles as a rapid low-cost methodology of anthropometric data collection for garment pattern development [15]. Other researchers developed the NTC Women’s Body Shape Analysis Tool (WBSAT), which used the hourglass shape as a launching point for understanding body shapes and identified three other shapes [10]. The new technology that includes digital virtual humans and digital virtual garments has had a substantial impact on the current apparel industry. Virtual simulation technology enables the visualization of a 3D virtual garment on a virtual avatar so that consumers can try on garments with their virtual mannequins before purchasing [26]. With the introduction of 3D scanning technology, it became possible to obtain more detailed dimensions, including items that were difficult to measure manually, such as depth and angles [49]. Previous studies have used 3D measurement data to analyze and classify the shapes of human bodies. The fashion industry is starting to use 3D body scanners to capture body measurements to make custom and tailored apparel. For instance, Simmons and Istook [44] developed software based on the Female Figure Identification Technique (FFIT) for apparel to differentiate the female population according to their body shapes using 3D body-scan data. They tested the software in nearly 700 subjects and identified nine body shapes that effectively represent the body types. Since it is very important to find the characteristics of each body shape among races and countries, several studies have been conducted about this direction. Body shape comparisons between countries provide the opportunity to discover ways of improving the sizing systems, as well as impact the development of international sizing standards that can have a considerable impact on brands producing products for a variety of international consumers with different sizes and shapes [24]. Newcomb (2006) classified the body shapes of Hispanic women in the U.S. using SizeUSA 3D measurement data and FFIT [38], and Masuda et al. [32] used 3D measurements to create body simulations to evaluate the shape images. Wells et al. [48] utilized 3D SizeUK and SizeUSA measurement data with indices for different body shapes to examine the size and shape differences between UK and US white adults. Vuruskan and Bulgun [47] developed an automatic system for numerically classifying body shapes, performing 3D body scans to take measurements and delineate body silhouettes. Song and Ashdown [46] examined the lower body shapes of the US female population using 3D SizeUSA body scan data. Lin et al. [27] compared the body measurements of citizens of four East Asian countries and identified differences among the four groups. Lee et al. [24] used FFIT to compare Size Korea and SizeUSA data and determine body shapes and body shape distributions of US and Korean women. Yi and Istook [50] also compared the body shapes of Korean and US women using ratios and indices from Size Korea and SizeUSA data.

However, it is important to emphasize that these devices can also lead to errors and present several disadvantages when compared with traditional physical measurement methods related to the type of technology (light, laser, or microwave) and how the image is collected [44]. In light-based systems, the color of the scanning gear, hair, and skin has a massive impact on the collection of the image. If there is too much of a difference between the scanning gear and the skin or the scanning gear is black or dark in color, then it is much more difficult to produce a good scanned image. Clothing worn during the scan process is an issue for both laser- and light-based scanning systems since both capture the surface of the garment over the outside of the body. Loose garments increase the dimensions of the measurements that are extracted, regardless of which system is used. In contrast, garments that are too tight are smaller measurements than they should be. Microwave systems are assumed to have no impact on the measurements obtained [44]. There are usually parts of the human body that cannot be ‘seen’ by the vision devices of the scanning system, such as the top of the head, the top of the shoulders, the bottom of the feet, the crotch at the junction of the legs, and the armpits depending on how many data-capturing devices there are in the system and their locations. The more sophisticated the system is, the more expensive it is. The consistency of measuring techniques between the scanners is a criticism. Among the growing number of scanners that are currently available, substantial variance exists in how each scanner captures or extracts specific body measurements [44].

## Materials and methods

As previously mentioned, the study was developed in two different steps. The first group of measurements aimed at the evaluation of the proposed system performances when observing regularly shaped objects of a known volume. In a second step, the proposed system was used to determine anthropometric parameters (linear measures and volumes) of the lower limbs of 8 subjects.

### Inanimate objects

**Fig. 1:**
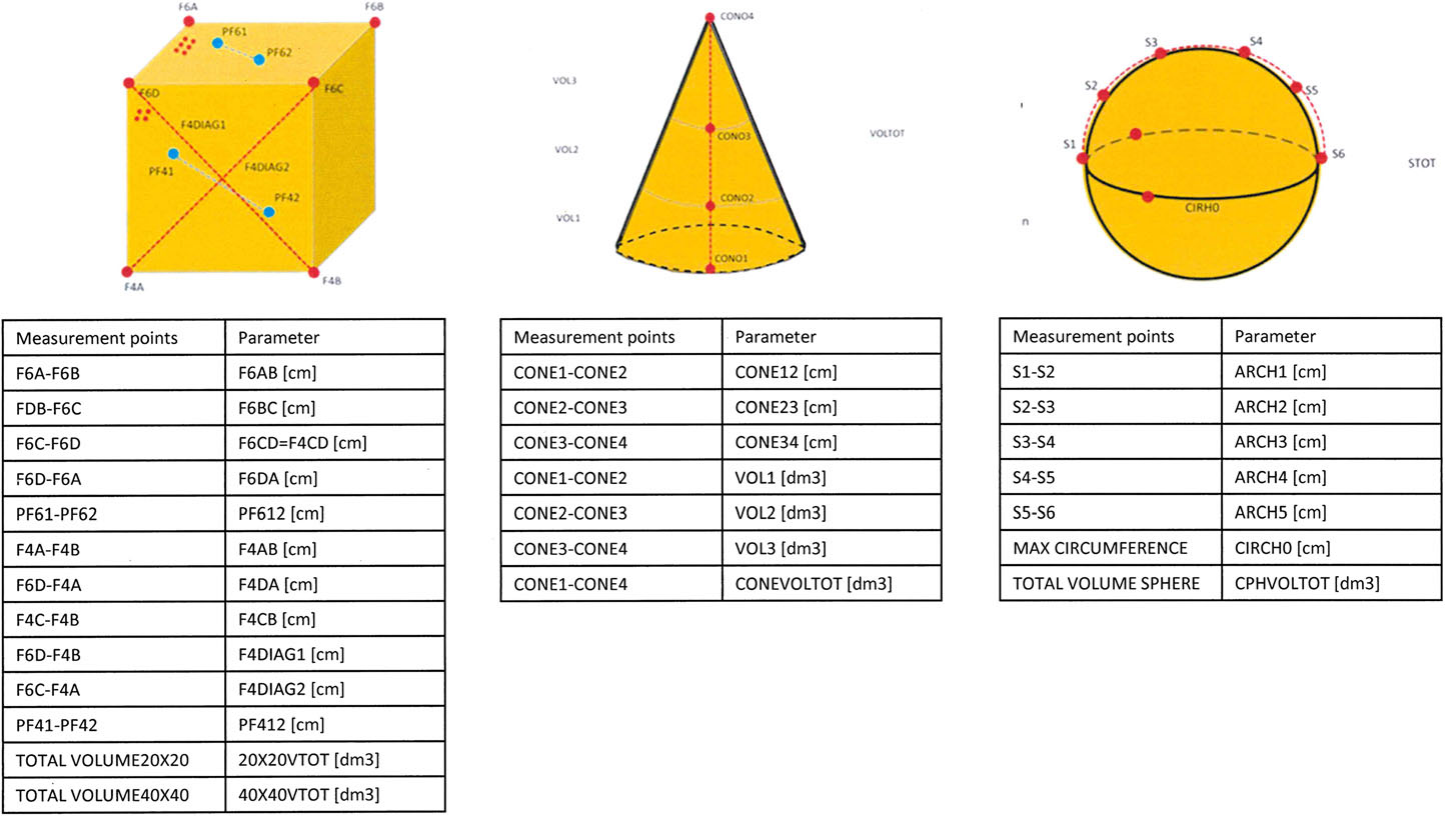
The objects and the measurements (linear and volumetric measures) for each object are reported. For the cube, the parameters are the same for the two cubes (20 cm and 40 cm in length)

The accuracy of the proposed system was assessed by observing four objects: two cubes (C1 and C2) with sides 20 cm and 40 cm in length, respectively, a cone 30 cm in height and a sphere with a diameter of 20 cm. The cone was marked at different heights, and measurements were taken at each length. In Fig. 1, the measured objects, the marked measurement points and the parameters for each object are displayed.

**Fig. 2:**
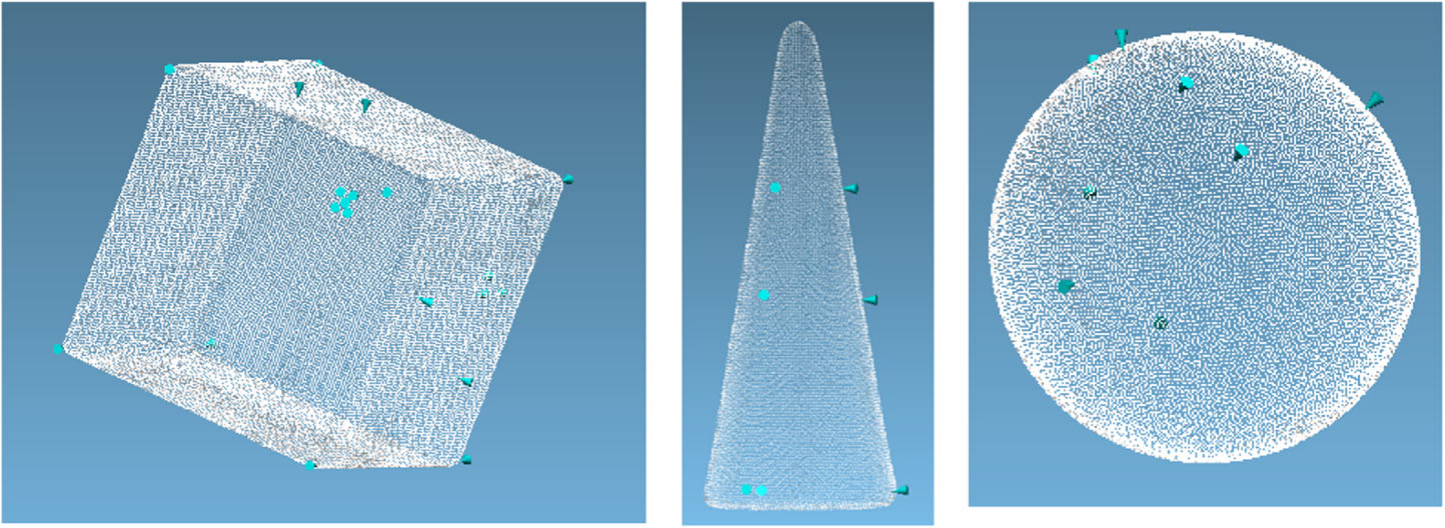
The point cloud for each scanned object. Igoodi: The gate

The reference values of the geometrical parameters were obtained with a hand-held LS3D system (O&P Scan Rodin4D, Pessac, France, laser peak power 1 mW, wavelength 670 nm, class I laser product). The scanner resolution is 0.1 mm, and the accuracy declared by the manufacturer is 0.75 mm. To reproduce the exact position of the measurement points indicated in Fig. 1, a laser pointer was used. Thus, a series of tags was created on the point cloud, which is defined as the set of points that represent the external surface of the scanned object (Fig. 2). The point cloud obtained by the measurement was analyzed offline with Rodin4D (version 5.6, Pessac, France). Each object volume was measured twice by the same operator.

**Fig. 3:**
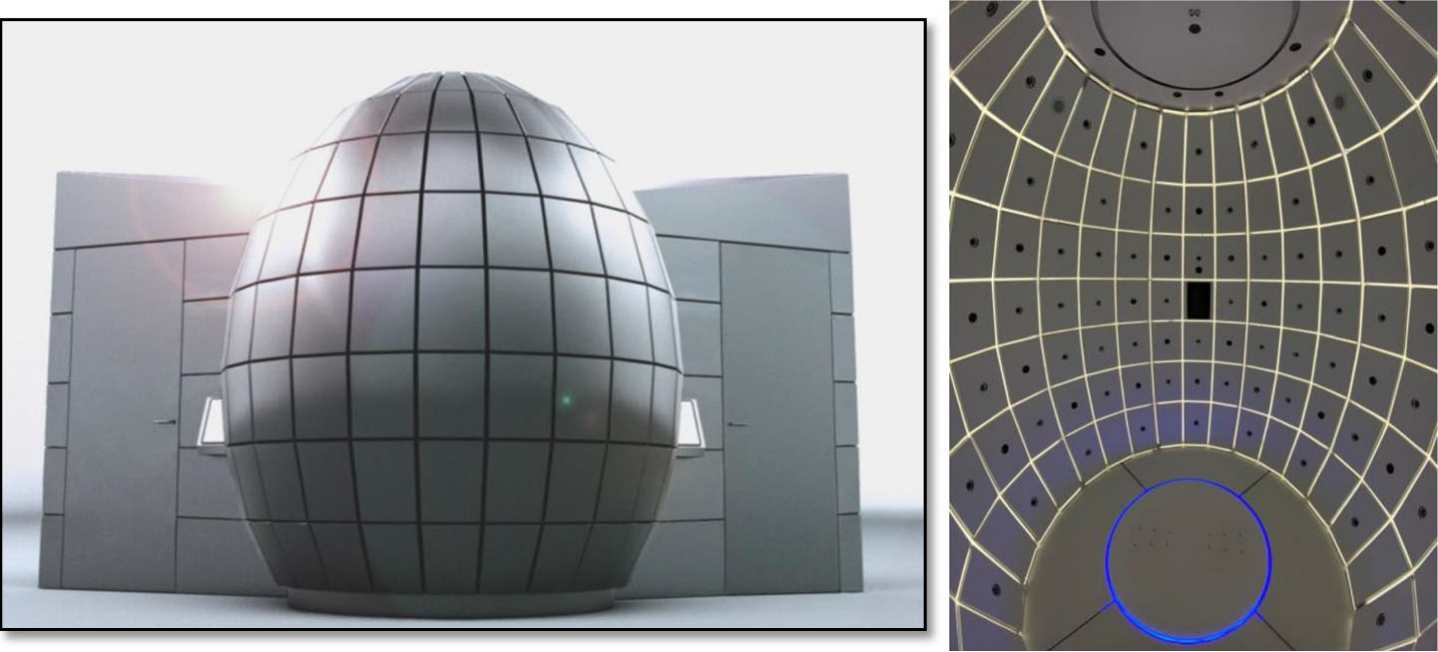
The Gate, as seen externally and internally

**Fig. 4:**
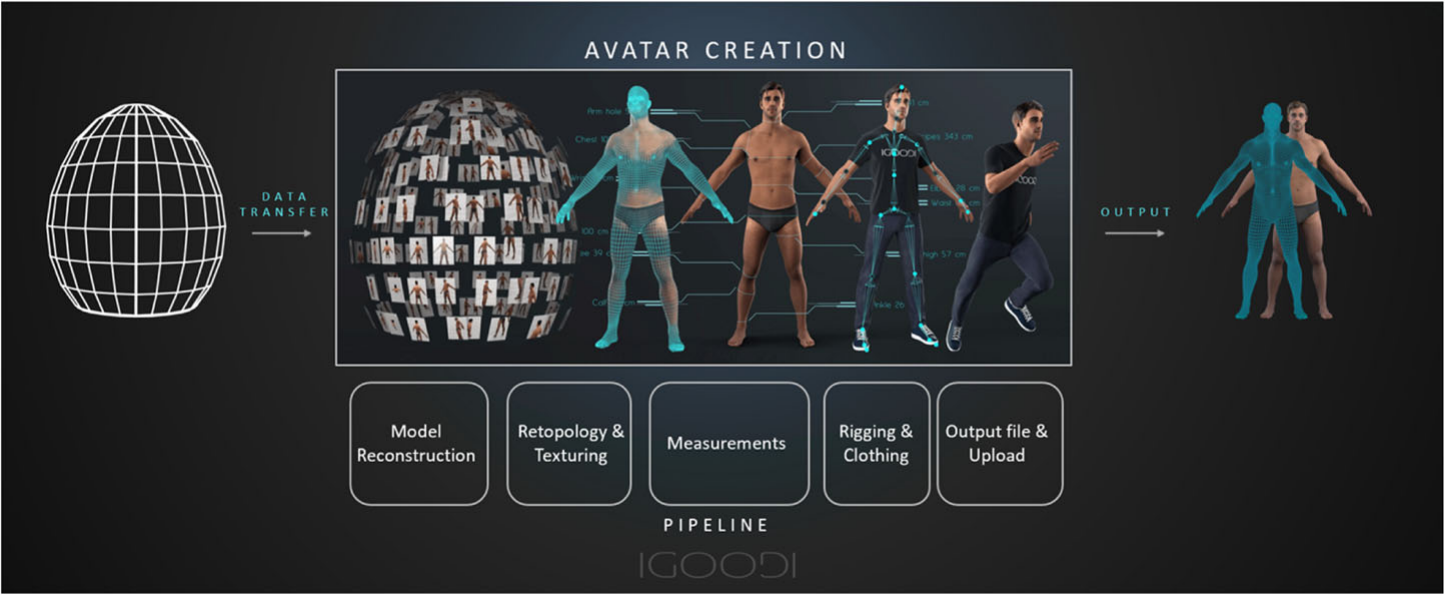
The end-to-end process, from scanning to production pipeline to the final outcome

The innovative proprietary scanning cabin described in this work (commercialized by IGOODI as “the Gate,” Fig. 3) is a photogrammetry-based 3D reconstruction studio that offers an autonomous scanning experience guided by a virtual avatar assistant to help through the process and without the need of a supervisor. The process entails a concurrent capture of 128 industrial cameras and sensors that capture height and weight. The height is captured via ultrasonic sensors connected to an Arduino that then send the data to the controlling software for calculating the height. The weight is captured by 2 × 2 load sensors that also send data via ethernet measurement to the Gate software. The procedure takes place within the body scanner cabin, which is completely enclosed for privacy purposes since the scanning is performed in underwear. The production process of the avatar is composed of various steps: the first step is the photogrammetry reconstruction of the 3D model based on the photos. The next steps are retopology, texturing and rigging, and finally transforming the 3D model into a Unity-compatible asset. The Gate and the production process are patented [5]. The following diagram (Fig. 4) depicts the steps of the pipeline that is also automatic and scalable (cloud architecture) to satisfy the scalability and mass avatar production requirement.

Although not in the scope of this paper, we describe the end-to-end production pipeline developed internally for reference. Overall, it is an automation process that encompasses all standard steps of a conventional manual pipeline to accommodate for scalability to mass production where there is no need to have 3D artists to perform those steps manually. Starting from the model reconstruction, which is based on photogrammetry, utilizing a command line-based automation batch processor and a commercially available photogrammetry suite^1^ utilizing structure from motion and multiview stereo reconstruction. The output of the first step is then passed onto the next one by the same batch processor, where the processing aims to apply a standard base mesh topology as per reference of standardized geometrical topology. This process uses surface mapping, nonrigid mesh registration and shape correspondence algorithms. At the same step, the texture projection is optimized for a better and cleaner fit on the model. As before, the output of this process is passed on by the batch processor to the next process, which has the mission of capturing a list of standard measurements commonly used in fashion, sports and nutrition. By using the common topology created during step 2, measurement positioning on each 3D model is standardized. At the successful completion of this step, automatic rigging and dressing follows, and finally, the output is fed into an uploader component that automatically passes on the assets to the secure cloud for storage and sharing with the user’s account and mobile device.

**Fig. 5:**
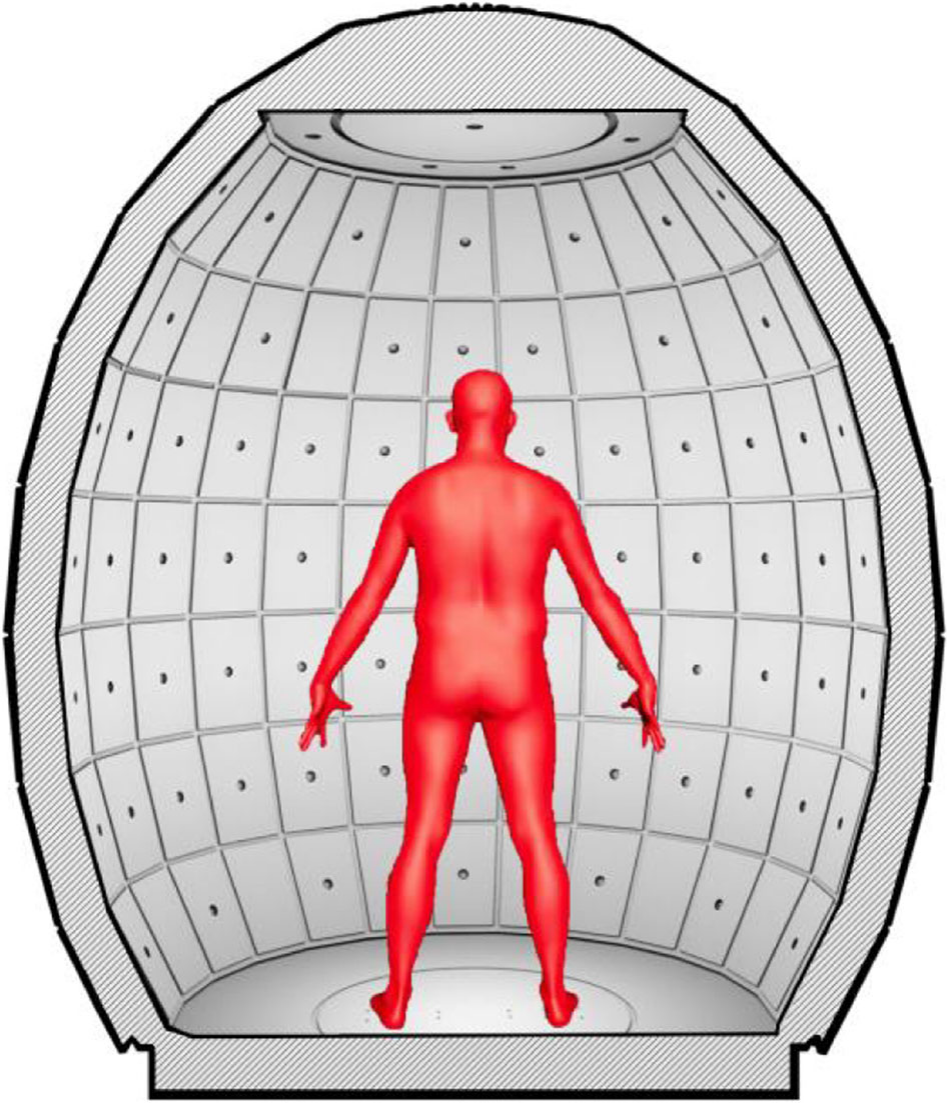
The scanning position and the A-Pose inside the Gate

The user follows the process presented by the avatar assistant inside the Gate and, during the various steps, is guided to assume specific poses that technically assure the best result for the scanning and body measurement, as well as the reconstruction of the 3D model and the eventual other steps of the production pipeline, such as rigging and skinning. The pose is forced through draft outlines that the user has to “fit” themselves into. These results assume the A-Pose (Fig. 5), a human body resembling the A letter, that is, with legs slightly apart and arms extended in parallel from the body and in, roughly, a 30–50-degree angle, depending on the subject.

**Fig. 6:**
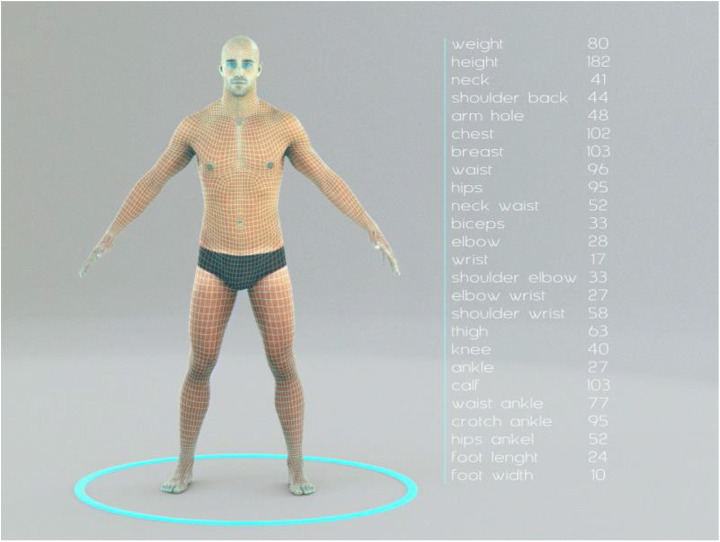
The avatar in the process and the list of anthropometric measurements

This process ensures the best pose for standard 3D reconstruction and compatibility with processes, such as rigging and skinning in the later processing stages. The outcome of the process is a fully rigged, highly accurate and realistic 3D model of the person along with the dataset of anthropometric measurements, as depicted in Fig. 6.

To validate the accuracy of the measurements through the Gate and LS3D, the comparison of the linear measurements with the gold standard, i.e., optoelectronic system, was performed for the cube with sides 20 cm in length. A motion capture system (SMART-DX, 8TVC, BTS, Milan, Italy) was used to acquire and reconstruct the 3D position of the markers placed on the point of reference of the cube used for LS3D scanning. Each measurement was performed once.

### Lower limb evaluation- pilot study

Eight normal-weight volunteers (4 males and 4 females; age: 37.6 + 10.3 years; body mass index: 22.8 + 3.2 kg/m^2^) were measured. The experimental procedure was explained in detail to the participants, and the study was carried out in accordance with the ethical standards of the Institute and with the 1964 Helsinki declaration and its latest amendments; written informed consent was obtained from the participants.

#### Laser scanning

**Fig. 7:**
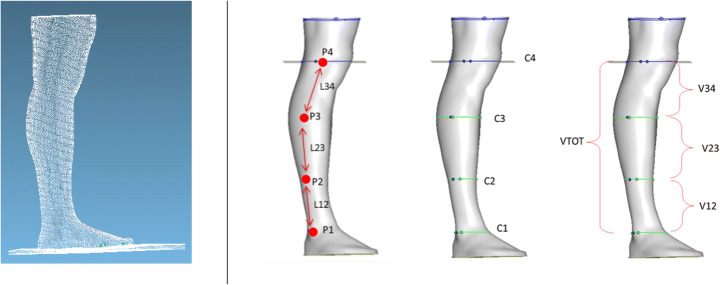
Point cloud for the leg and the 3D scanning parameters of the leg. P1: external malleolus; P2: middle point between fibula head and malleolus; P3: fibula head, P4: center of the knee; L: linear measure; C: circumference measure; V: volume; VTOT: total volume

The LS3D system previously described was used. To guarantee the proper accuracy during the scanning phase, it was necessary to ensure that the subjects could maintain a stable position for the entire measurement duration, which was, in general, 2–3 minutes; to achieve this stability, participants were standing up. The leg was defined from the center of the knee to the malleolus, and it was divided into three parts (Fig. 7).

To develop the clinical scanning protocol, three types of parameters were defined: linear measures, circumferential measures and volume measures, indicated with L, C and V, respectively. All subvolumes related to the linear and circumferential points were defined and computed. The reference points were identified manually, and they were the following: P1: external malleolus; P2: middle point between fibula head and malleolus; P3: fibula head; P4: center of the knee, i.e., femoral epicondyle. The leg length, computed as the distance between P1 and P4, was divided into three parts. The corresponding linear measures and circumferences were defined, and the subvolumes (V12, V23 and V34 parameters) were calculated. The total volume (VTOT parameter) was computed as the sum of the three subvolumes [9].

Bilateral measurements were performed three times for each subject with both methods (LS3D and the Gate system) and compared to verify possible errors and differences. Thus, a total of 48 measurements of LS3D and the Gate system were each produced.

#### Statistical analysis

The Kolmogorov-Smirnov test was used to verify if the parameters were normally distributed. The data were normally distributed so the mean and the standard deviation were calculated for each parameter.

For the analysis of the first study group (inanimate object), the variability between measurements was assessed using the standard deviation; the accuracy was assessed according to the following formula [Eq. 1]

1 $$ accuracy=\frac{measured\ value- actual\ value}{actual\ value} $$

where the actual value is the measurement value by using the optoelectronic system and the measured value is the measurement value by using the Gate or LS3D.

For the second study group (human study), the correlation between the Gate and LS3D measures was analyzed, and Pearson’s correlation coefficient was calculated. To evaluate the level of agreement (LOA) between the two methods, a Bland–Altman plot was performed [3]. This is a graphical method for comparing two measurements of the same variable where the X-axis represents the mean of two measurements, and the Y-axis represents the difference between. The plot can then highlight anomalies. For example, if one method always gives a result that is too high, then all points are above or below the zero line. This can also highlight that one method overestimates high values and underestimates low values. Otherwise, if the points on the Bland-Altman plot are scattered all over the place above and below zero, then it suggests that there is no consistent bias of one approach versus the other.

The intraclass correlation coefficient (ICC) was used to verify the relative reliability, which was deemed acceptable if the ICC statistic was greater than 0.71 [4], while the typical error of measurement (TEM) was used to verify the absolute reliability (TEM) [4]. Statistical differences between the Gate and LS3D were evaluated using a t-test. The accuracy was assessed according to Eq. 1, where the actual value was the measurement value by using LS3D and the measured value was the measurement value by using the Gate system. The null hypothesis was rejected when probabilities were below 0.05 (p < 0.05).

## Results

### Inanimate objects

Linear and volume measurements on all objects are summarized in Table 1. The measured variability, quantified by the standard deviation, is generally lower when the measurements are performed by the Gate system than by the LS3D system.

**Table 1:** Measurement (linear and volume measures) of the objects (mean and standard deviation)

	The Gate	LS3D
*Cube 20 cm*		
F6AB [cm]	19.5 (0.00)	19.45 (0.07)
F6BC [cm]	19.6 (0.00)	19.05 (0.07)
F6CD=F4CD [cm]	19.5 (0.00)	19.35 (0.07)
F6DA [cm]	19.4 (0.00)	19.3 (0.14)
PF612 [cm]	4.923 (0.04)	5.15 (0.21)
F4AB [cm]	19.7 (0.00)	19.45 (0.07)
F4DA [cm]	19.3 (0.00)	19.15 (0.21)
F4CB [cm]	19.3 (0.00)	19.1 (0.00)
F4DIAG1 [cm]	27.6 (0.00)	27.3 (0.00)
F4DIAG2 [cm]	27.55 (0.07)	27.45 (0.07)
PF412 [cm]	11.31 (0.01)	11.25 (0.07)
20x20VTOT [dm3]	7.7935 (0.01)	7.74 (0.01)
*Cube 40 cm*		
F6AB [cm]	39.15 (0.07)	39.45 (0.49)
F6BC [cm]	39.05 (0.07)	39.15 (0.21)
F6CD=F4CD [cm]	38.8 (0.14)	38.1 (0.85)
F6DA [cm]	38.45 (0.07)	38.95 (1.20)
PF612 [cm]	8.055 (0.08)	8.25 (0.35)
F4AB [cm]	39.35 (0.07)	38.9 (0.57)
F4DA [cm]	39.2 (0.00)	39.4 (0.57)
F4CB [cm]	39.4 (0.00)	39.1 (0.71)
F4DIAG1 [cm]	55.5 (0.00)	55.4 (0.14)
F4DIAG2 [cm]	55.35 (0.07)	55.7 (0.42)
PF412 [cm]	26.125 (7.11)	21 (0.00)
40x40VTOT [dm3]	63.4 (0.00)	63.585 (0.49)
*Cone*		
CONE12 [cm]	12.1 (0.00)	12.1 (0.00)
CONE23 [cm]	6.8 (0.00)	6.9 (0.14)
CONE34 [cm]	10.4 (0.14)	10.45 (0.07)
VOL1 [dm3]	0.954 (0.00)	0.875 (0.01)
VOL2 [dm3]	0.22 (0.00)	0.22 (0.00)
VOL3 [dm3]	0.103 (0.00)	0.61 (0.71)
CONEVOLTOT [dm3]	1.277 (0.00)	1.30 (0.01)
*Sphere*		
ARCH1 [cm]	4.81 (0.01)	4.95 (0.07)
ARCH2 [cm]	5.725 (0.02)	5.75 (0.21)
ARCH3 [cm]	5.805 (0.01)	6.1 (0.28)
ARCH4 [cm]	6.86 (0.03)	7.05 (0.35)
ARCH5 [cm]	7.225 (0.02)	6.95 (0.35)
CIRCH0 [cm]	62.86 (0.35)	62.35 (0.07)
SPHVOLTOT [dm3]	4.153 (0.00)	4.125 (0.01)

The linear measures of the cube with sides of 20 cm measured by the Gate and LS3D systems were plotted against the actual measures as measured through the optoelectronic system. Both the Gate and LS3D systems have maximum errors that are lower than 0.7 cm. However, the values reported in Table 2 show that the accuracy of the measures obtained with the Gate is better than that of the LS3D when measuring the cube with sides of 20 cm.

**Table 2:** Values of accuracy of the measures with the Gate and LS3D systems for the cube with sides of 20 cm in length with respect to the gold standard, i.e., the optoelectronic system

	Accuracy, the Gate	Accuracy, LS3D
F6AB [cm]	−1.52%	−1.52%
F6BC [cm]	−1.01%	−4.04%
F6CD=F4CD [cm]	−1.52%	−2.02%
F6DA [cm]	0.00%	−1.03%
PF612 [cm]	−1.50%	0.00%
F4AB [cm]	−1.99%	−2.99%
F4DA [cm]	−0.52%	−0.52%
F4CB [cm]	−1.03%	−2.05%
F4DIAG1 [cm]	−1.08%	−2.15%
F4DIAG2 [cm]	−1.25%	−1.43%
PF412 [cm]	0.09%	0.00%
20x20VTOT [dm3]	–	–

### Lower limb evaluation- pilot study

The linear, circumferential and volume measures related to the legs of the participants are summarized in Table 3.

**Table 3:** Values (mean and standard deviation) of the linear, circumferential and volume measures on the assessed legs with the Gate and LS3D systems. * = p < 0.05, Gate vs. LS3D (after the t-test comparison). The Pearson coefficient value (r value) of the correlation between the IGOODI and LS3D systems; all the correlations are statistically significant (p < 0.05)

	The Gate	LS3D	Accuracy	Root mean squared error	Pearson coefficient value
L12 [cm]	12.79 (1.74)	12.62 (1.40)	1.33%	0.265	0.8621
L23 [cm]	14.12 (0.94)	14.10 (0.94)	0.13%	0.267	0.919
L34 [cm]	14.59 (0.91)	14.66 (1.07)	−0.30%	0.624	0.811
C1 [m]	0.27 (0.02)	0.28 (0.02)	−0.67%	0.012	0.864
C2 [m]	0.26 (0.02)	0.27 (0.02)*	−2.08%	0.009	0.923
C3 [m]	0.37 (0.02)	0.37 (0.02)*	−1.17%	0.009	0.940
C4 [m]	0.38 (0.03)	0.38 (0.03)	−1.09%	0.018	0.796
V12 [dm3]	0.31 (0.07)	0.56 (0.12)*	−44.71%	0.038	0.973
V23 [dm3]	0.93 (0.19)	1.19 (0.19)*	−22.81%	0.043	0.978
V34 [dm3]	0.95 (0.18)	1.46 (0.26)*	−35.28%	0.045	0.985
VTOT [dm3]	2.30 (0.63)	3.22 (0.26)*	−29.17%	0.093	0.986

**Fig. 8:**
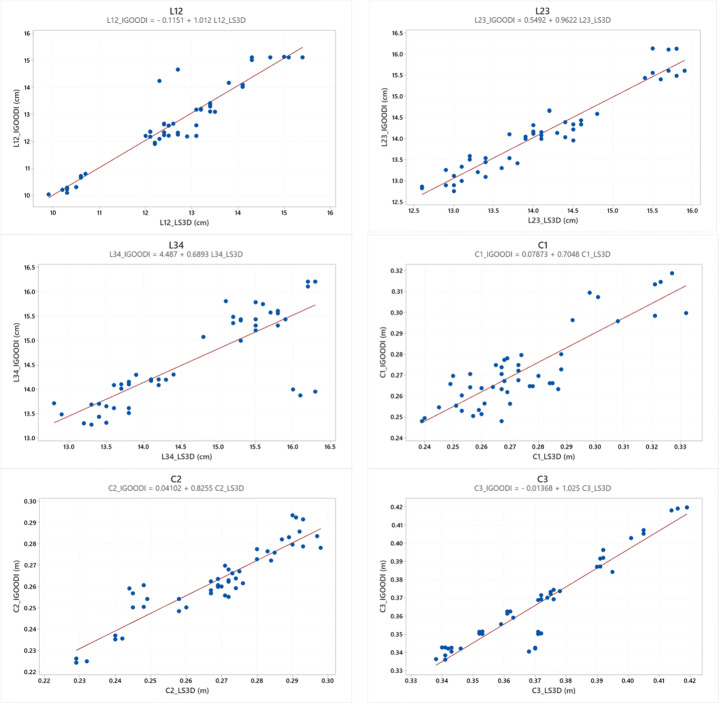

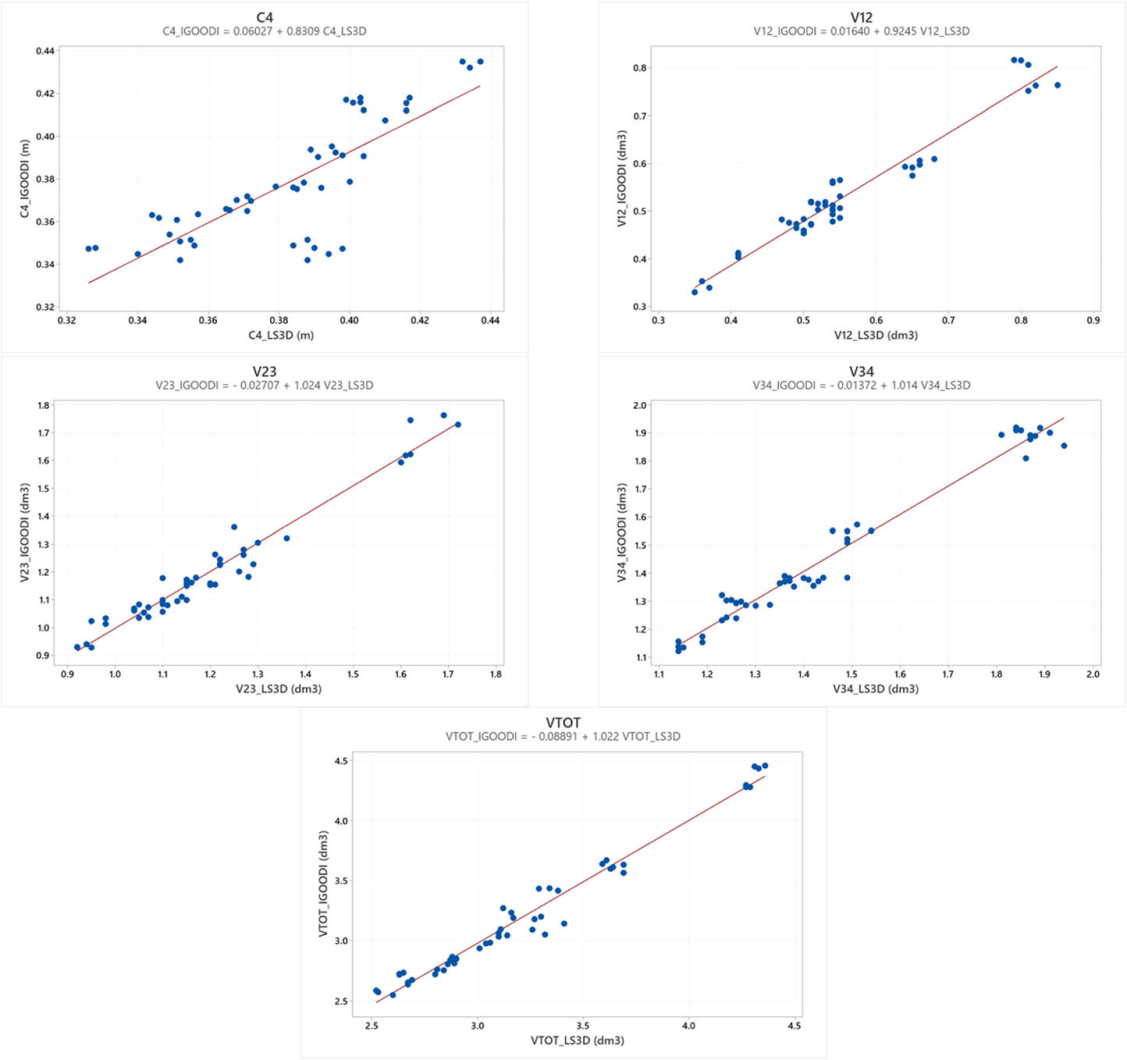
Values of the measured parameters (the Gate vs. LS3D) of the evaluated sample with the linear relationship equations for each graph

The Pearson coefficient values of the correlation between the measures determined from the Gate and LS3D systems were all statistically significant (p < 0.05) and highlight the validity of the proposed approach. These results indicate that the correlations are statistically significant; thus, there is a positive relationship between the two methods for all the parameters. However, it is not possible to affirm that there is a total agreement between the two methods, as the points do not perfectly lie along the line of equality, as presented in Fig. 8. Figure 8 represents the trend of the measured parameters (the Gate vs. LS3D) of the evaluated sample. Each dot represents the comparison between the two methods (i.e., 8 subjects, right and left limb assessed three times, in total 48 dots).

**Fig. 9:**
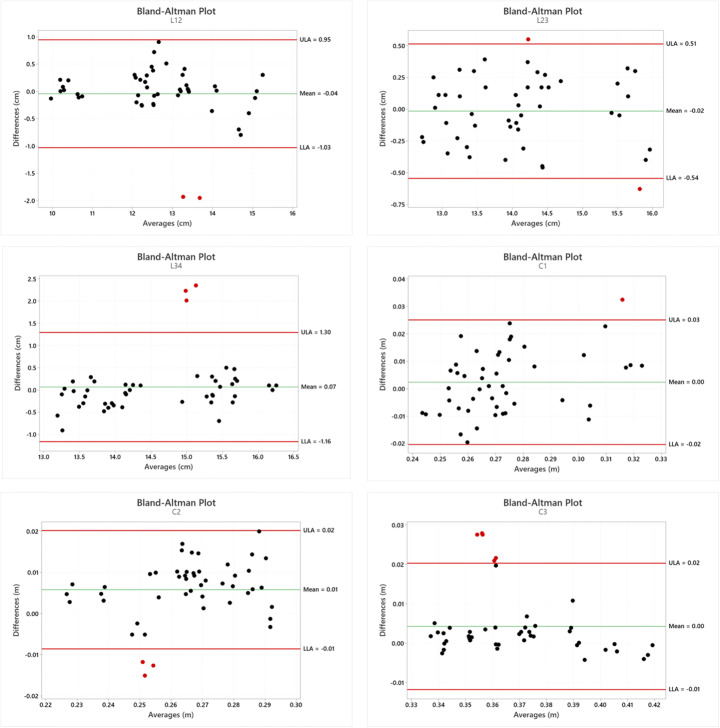

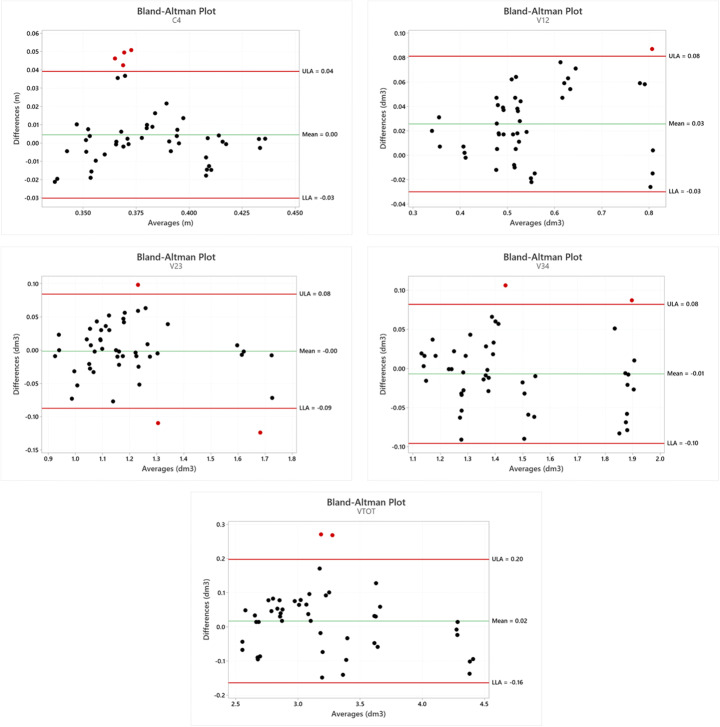
Bland-Altman plots using the Gate and LS3D systems plotted against the difference between the two methods for the considered parameters

In Fig. 9, the Bland-Altman plot is displayed for each considered parameter. The mean difference is close to zero in most measurements, and it is possible to observe that globally, there is an accordance between the two methods even if in each graph some measurements are not in complete agreement. The considerations related to the Bland-Altman plots can be considered coherent with the values of accuracy between the two systems displayed in Table 3 and Fig. 7.

The reliability results of both methods are presented in Table 4.

**Table 4:** Relative (ICC) and absolute (TEM) reliability of the two methods. ICC, intraclass correlation coefficient; TEM, typical error of measurement

	LS3D		The Gate	
	ICC	TEM	ICC	TEM
L12	0.995	0.93	0.842	0.87
L23	0.979	0.95	0.999	0.96
L34	0.981	0.91	0.998	0.93
C1	0.967	0.02	0.974	0.02
C2	0.992	0.02	0.989	0.02
C3	0.998	0.02	0.999	0.02
C4	0.991	0.03	0.997	0.03
V12	0.997	0.12	0.998	0.12
V23	0.993	0.20	0.998	0.21
V34	0.996	0.26	0.999	0.27
VTOT	0.998	0.53	0.999	0.55

## Discussion and conclusion

The objective of this study is to verify the accuracy and precision of the measurement capability of the Gate technology to reproduce precise reconstructions of objects, including segments of the human body, by comparing between the Gate system and the laser scanner three-dimensional method on a series of regularly shaped objects of known volume. Our results show that both the Gate and LS3D systems are extremely close to the actual measurements of the objects, as evidenced by the coefficient values of the correlation between measurements determined from the Gate and LS3D systems, which were all statistically significant. In addition, it is important to emphasize that when the accuracy of the measurements with the Gate and LS3D was assessed with respect to the gold standard (optoelectronic system), the Gate measurements seemed to be more accurate than LS3D technology. The results obtained in terms of the linear measures of the cube with sides of 20 cm measured by the Gate and LS3D system do in fact show that the accuracy of the measurements obtained with the Gate is better than those of the LS3D.

The evaluations of the human lower limbs show that the linear measurements, diameters and volumes assessed with the Gate system and LS3D present a general agreement between the two methods, and their consistency decreased from linear to volumetric measurements.

Our results show that the Gate technology can represent an accurate and reliable tool for volume measurement and has strong potential in terms of human body reconstruction. Such avatars, beyond the 3D realistic representation, contain a series of anthropometric data as precise body measurements. This concept, coined the smart body, renders avatars enablers of a wide range of use cases of impact in the fields of fashion, wellness and healthcare.

In this context, the use of an accurate and reliable tool for volume measurement is very important, and the use of technology based on three-dimensional body-scanning technologies is having a growing impact on the product development, production and consumption processes. However, in retail, there are some criticisms, and among the growing number of scanners that are currently available, great variance exists in how each scanner captures or extracts specific body measurements [44]. Recently, an automatic process to capture measurements and an ecosystem of technologies that enable avatars to become smart bodies by enriching their data in a continuous manner have been developed.

Based on the accuracy level of the Gate shown in this study, avatars can enable the online shopper to be confident in their size selection as a facility offered by the smart body concept. Therefore, for the use case of fashion, the approach can be deemed applicable and of satisfactory accuracy for sartorial purposes. The Gate can be easily installed within malls and physical stores, where in a very short time, the user can have their own smart body, and thus, enable the size suggestion facility, as well as remote measurements. The derivative applications of avatars and the smart body concept in the fashion industry are envisaged to substantially reduce the returns of goods bought online and eventually increase the eco-sustainability of fashion with reduced transport and materials. Furthermore, with the highlighted importance during the COVID-19 pandemic, remote online solutions such as the smart body concept presented in this study, enable a sustainable economy that is resilient to imposed restrictions of various kinds. The same indication also applies to the other verticals mentioned in the introduction section of this paper, namely, wellness with nutrition and personal training as smart body services, in as much more accuracy demanding areas, such as medical applications (orthopedics, dermatology). The user in this case can engage with wellness professionals (nutritionists, personal trainers) remotely and have access to a highly personalized service prescribed on their avatar. Furthermore, remote consistent monitoring of the performance and outcomes of the prescribed wellness regimes can be accessible through periodic scans and comparisons with previous states. Those use cases demonstrate the limitation of this study and the further need for follow-up studies with a broader population and demographic data to further enhance the confidence of applicability of the technical accuracy to each use case. However, it is safe to conclude that the concept of Smart the Body hypothesized in this study as containers of anthropometric and personal data is applicable and can provide strong advantages and opportunities for disruption in a diverse range of sectors, while having a positive impact on sustainability and resource efficiency and offer opportunities for new sectors to arise in the form of an avatar economy.

In conclusion, the current paper presents a scalable solution for the mass production of avatars with accurate anthropometric data to become the foundation for a digital ID for web3 and the metaverses looming as the next major evolution in technological advances.

## Limitations and future work

This paper contains a study of accuracy as its baseline versus a gold standard from a technological point of view (laser technology). However, it presents some limitations. First, future studies can be conducted to compare the measurements resulting from the Gate vs. those obtained by an anthropometrics specialist, as in the literature [22, 23, 34].

Another limitation of this current work is that although it presents a series of potential use cases that have a requirement accuracy in body scanning and measurement, it focuses mainly on the one that has the highest tolerance for error (fashion), with plans to evaluate more rigorously and with more appropriate research methods the applicability and utility in the areas of health and wellness. For example, to evaluate the derivative applicability in a potential postural analysis, the accuracy of measurements on its own would not satisfy the requirement. Therefore, future research will be conducted to expand the numbers, demographics and variability of validating a broader range of body parts and measurements.

## Data Availability

The datasets generated and analysed during the current study are available from the corresponding author on reasonable request.

## Footnotes

1 Reality Capture: https://www.capturingreality.com/
